# Accuracy of clinical diagnosis in neurodegenerative diseases - a study of 455 autopsy cases

**DOI:** 10.1007/s00415-026-13680-w

**Published:** 2026-02-20

**Authors:** Jonathan Vöglein, Thomas Arzberger, Irena Ebner, Jochen Herms, Sigrun Roeber, Viktoria Ruf, Adrian Danek, Armin Giese, Günter U. Höglinger, Johannes Levin

**Affiliations:** 1 Department of Neurology, LMU University Hospital, LMU Munich, Marchioninistraße 15, 81377 Munich, Germany; 2 German Center for Neurodegenerative Diseases (DZNE), Feodor-Lynen-Straße 17, 81377 Munich, Germany; 3 Munich Cluster for Systems Neurology (SyNergy), Feodor-Lynen-Straße 17, 81377 Munich, Germany; 4 Center for Neuropathology and Prion Research, Faculty of Medicine, LMU Munich, Feodor-Lynen-Straße 17, 81377 Munich, Germany; 5 Department of Psychiatry and Psychotherapy, Ludwig-Maximilians-Universität München, Nussbaumstraße 7, 80336 Munich, Germany; 6 MODAG GmbH, Mikro-Forum-Ring 3, 55234 Wendelsheim, Germany

**Keywords:** Neurodegenerative diseases, Clinical diagnosis, Neuropathology, Diagnostic accuracy, Clinical–neuropathological correlation

## Abstract

**Background:**

Precision of clinical diagnosis in neurodegenerative diseases is critically important for clinical care and study recruitment. This study aimed to investigate the clinical accuracy using gold-standard neuropathological reference.

**Methods:**

Neuropathological diagnoses from the Neurobiobank München were correlated with real-world clinical diagnoses from hospitals in Germany. Accuracy metrics, including sensitivity, specificity, and area under the curve (AUC) of clinical diagnoses, were calculated.

**Results:**

Among nine neuropathologically diagnosed neurodegenerative diseases (Alzheimer’s disease, argyrophilic grain disease, corticobasal degeneration, frontotemporal lobar degeneration, Huntington’s disease, Lewy body disease, motor neuron disease, multiple system atrophy, and progressive supranuclear palsy) with a total of 455 cases, clinical sensitivity varied widely (0–100%) whereas specificity was consistently high (89.5–100%). Accuracy was very good (AUC > 0.9) for Huntington’s disease, motor neuron disease, and multiple system atrophy; good (AUC = 0.8–0.9) for Alzheimer’s disease, dementia with Lewy bodies/Parkinson’s disease, and progressive supranuclear palsy; moderate (AUC = 0.7–0.8) for frontotemporal dementia, limited (AUC = 0.51–0.7) in the *n* = 20 cases with corticobasal degeneration, and no discriminatory capacity (AUC = 0.5) in the *n* = 6 cases with argyrophilic grain disease.

**Conclusions:**

Clinical diagnostic accuracy of neurodegenerative diseases varies, with sensitivity as the main limiting factor. Improving diagnostic sensitivity will be essential for early and accurate patient identification, especially as disease-modifying therapies targeting causal proteinopathies become available. Achieving this will depend on the development and clinical implementation of reliable molecular biomarkers that indicate the causal proteinopathies of neurodegenerative diseases.

## Introduction

Pattern and distribution of proteinopathies are used to define and diagnose neurodegenerative diseases [1]. The full extend, spatial distribution, cellular localization, diversity, and co-occurrence of protein pathologies can only be assessed comprehensively by neuropathological examination. This may be the reason why discrepancies between clinical and neuropathological diagnosis were reported in Alzheimer’s disease (AD) [2] and Lewy body disease (LBD) [3, 4]. These discrepancies are also true for frontotemporal lobar degeneration (FTLD) [5], multiple system atrophy (MSA), progressive supranuclear palsy (PSP), and in particular corticobasal degeneration (CBD) [6, 7]. Whereas clinical precision in the aforementioned neurodegenerative diseases has been investigated in single studies and in part without neuropathological examination as the reference, no efforts have been made to evaluate the accuracy of clinical diagnosis in motor neuron disease (MND), Huntington’s disease (HD), and argyrophilic grain disease (AGD) using post-mortem validation.

To make effective disease-modifying treatments that target the causal proteinopathies available to patients, its crucial to identify the various neurodegenerative diseases in clinical settings with accuracy. Therefore, we set out to investigate within a single study how precise clinicians diagnose within a wide spectrum of neurodegenerative diseases (AD, AGD, CBD, FTLD, HD, LBD, MND, MSA, and PSP) in real-world settings, and where improvements are needed to successfully identify patients with neurodegenerative diseases for potential causal treatment. For referencing clinical diagnosis to standardized neuropathological evaluation, we leveraged the Neurobiobank Munich, a Brainbank embedded in Brain NetEurope [8] holding a wide spectrum of different neurodegenerative diseases with large numbers of cases.

## Methods

### Participants

All 1310 autopsy cases between 1993 and 2017 from the Neurobiobank München (NBM) (Munich, Germany) at Ludwig-Maximilians-Universität (LMU) Munich were screened for neurodegenerative diagnoses. Neuropathological diagnoses that occurred in at least five cases were included in the study. Only cases with sufficient clinical information regarding clinical diagnoses were considered.

All participant brain donations were obtained after acquiring written informed consent from participants and/or their legal representatives in accordance with applicable local laws and practices. The Institutional Review Board of the LMU Munich, Germany, approved this study (project number: 17–507).

### Neuropathological assessment

NBM cases were assessed using established procedures with a standardized protocol, following the Code of Conduct of BrainNet Europe [8]. Neuropathological diagnoses were made according to current diagnostic criteria [9–25]. All neuropathological evaluations were made by two senior neuropathologists experienced in neurodegenerative diseases in consensus.

In 8 of 9 clinical MND diagnoses that were followed by a neuropathological diagnosis of FTLD, neuropathologically also MND was present. There were 6 neuropathological MND-TDP diagnoses. Two cases showed also FLTD. These results reflect the FTLD/MND spectrum of TDP43 diseases. For this study, each case was assigned a single predominant neuropathological diagnosis determined in consensus based on the dominant proteinopathy and its distribution.

### Clinical diagnoses

Real-world clinical diagnoses were extracted from medical records of admitting hospitals in Germany. Real-world clinical diagnoses were made through the physicians from the admitting hospitals and based on patient history, physical examination, diagnostic tests, clinical judgment, diagnostic criteria, and/or diagnostic guidelines. Clinical diagnoses were correlated with neuropathological diagnoses. If different clinical diagnoses were made in consecutive work-ups, the accurate diagnosis was taken if made; otherwise, the last available diagnosis was used for analysis.

As CBD, now a solely neuropathological diagnosis [11], was used also as clinical diagnosis until the mid-to-late 2010s and all but one (CBS) diagnosing physician used this term for the clinical diagnoses included in this study, we stayed with CBD for clinical diagnosis as it was the actual real-world clinical diagnosis.

### Statistical analysis

Sensitivity, specificity, and discriminatory power of clinical diagnoses for the respective correct neuropathological diagnosis were calculated using receiver-operating characteristic (ROC) curves and area under the curve (AUC) analysis. P values for testing the null hypothesis AUC = 0.5 were calculated. For the present analysis, each case was assigned a single predominant neuropathological diagnosis. Predominance was determined in consensus based on the dominant proteinopathy and its distribution. Relevant co-pathologies (e.g., concomitant AD, Lewy body, or TDP‑43 pathology) were documented in the neuropathology reports but were not modeled in the primary accuracy analyses.

Clinical and neuropathological population characteristics were compared between groups using t test for continuous and Chi-square test for categorical variables.

All tests were performed two-sided. P values less than 0.05 were considered statistically significant. IBM SPSS Statistics Version 29 was used for statistical analyses.

## Results

### Participants

From the 1310 cases in the Neurobiobank Munich, 455 cases with a neuropathological diagnosis of a neurodegenerative disease and with available valid clinical information were included in the study. Table 1 summarizes characteristics of the study population.

**Table 1:** Clinical and neuropathological characteristics of study participants

	AD (*n* = 132)	AGD (*n* = 6)	CBD (*n* = 20)	FTLD (*n* = 47)	HD (*n* = 22)	LBD (*n* = 104)	MND (*n* = 10)	MSA (*n* = 37)	PSP (*n* = 77)	Total (*n* = 455)
Survival, mean years [SD], *n*	9.3 [5.2], 54	na, 1	6.5 [3.3], 10	4.5 [13.2], 29	15.0 [6.8], 9	10.9 [7.9], 30	3.2 [2.0], 9	7.5 [3.2], 21	7.9 [4.6], 55	8.4 [7.9], 218
Female sex, *n* [%]	71 [54]	1 [17]	8 [40]	15 [33]	10 [48]	43 [41]	3 [30]	18 [49]	41 [54]	210 [47]
Age at onset, mean years [SD], *n*	62 [13], 54	na, 1	58 [7], 10	61 [12], 29	45 [14], 9	67 [10], 30	61 [12], 9	58 [9], 21	64 [8], 55	61 [12], 218
Age at death, mean years [SD], *n*	71 [13], 54	na, 1	65 [7], 10	65 [10], 29	60 [13], 9	77 [6], 30	64 [12], 9	66 [8], 21	72 [7], 55	70 [10], 218
Disease duration @ first clinical diagnosis, mean years [SD], *n*	3.6 [3.4], 52	na, 1	4.1 [3.8], 10	3.2 [4.8], 28	9.0 [6.2], 8	3.3 [6.1], 29	1.4 [1.2], 8	2.8 [2.7], 20	3.5 [5.5], 53	3.8 [5.5], 209
Disease duration @ correct clinical diagnosis, mean years [SD], *n*	3.8 [3.6], 52	na, 1	5.3 [5.2], 10	4.7 [5.5], 28	9.0 [6.2], 8	5.8 [8.3], 29	1.4 [1.2], 8	4.7 [3.6], 20	3.8 [3.4], 53	4.5 [4.8], 209

Numbers of cases with available data for the respective parameters are included in the table. Sex was available for all cases.

*AD* Alzheimer disease, *AGD* Argyrophilic grain disease, *CBD* corticobasal degeneration, *FTLD* frontotemporal lobar degeneration, *g* gramm, *HD* Huntington’s disease, *LBD* Lewy body disease, *MND* motor neuron disease, *MSA* multiple system atrophy

### Neuropathological and clinical diagnoses

Neuropathological diagnoses comprised AD (*n* = 132), AGD (*n* = 6), CBD (*n* = 20), FTLD (*n* = 47; FTLD-TDP: *n* = 38, FTLD-Tau (Pick’s disease): *n* = 5; FTLD-FUS: *n* = 4), HD (*n* = 22), LBD (*n* = 104), MND (*n* = 10), MSA (*n* = 37), and PSP (*n* = 77).

Clinical diagnoses in these cases included AD (*n* = 136), CBD (*n* = 17), Creutzfeldt–Jakob disease (CJD, n = 6), dementia with Lewy bodies (DLB, n = 11), frontotemporal dementia (FTD, *n* = 44), HD (*n* = 23), MND (*n* = 20), MSA (*n* = 36), Parkinson’s disease (PD, *n* = 92), PSP (*n* = 60), and vascular dementia (VD, *n* = 10).

**Fig. 1:**
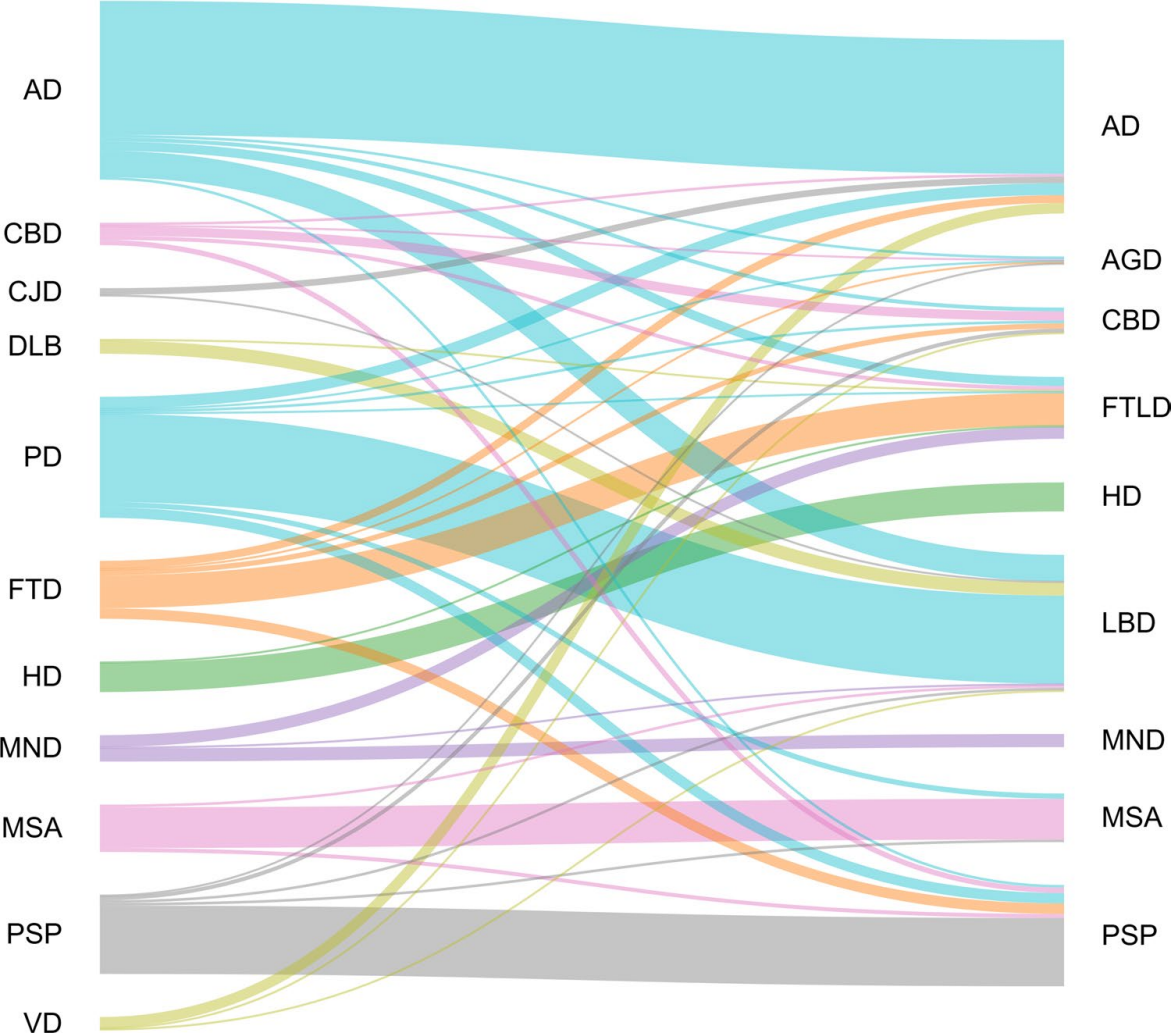
Relationship between clinical (left) and neuropathological diagnoses (right). Description: As CBD, now a solely neuropathological diagnosis, was used also as clinical diagnosis until the mid-to-late 2010 s and all but one (CBS) diagnosing physician used this term for the clinical diagnoses included in this study, we stayed with CBD for clinical diagnosis as it was the actual real-world clinical diagnosis. In 8 of 9 clinical MND diagnoses that were followed by a neuropathological diagnosis of FTLD, neuropathologically also MND was present. For this study, the predominant protein pathology and its predominant distribution determined the neuropathological diagnosis. That is, the subset of clinical MND diagnoses that resulted in a neuropathological diagnosis of FTLD may not be due to clinical inaccuracy. Patients with the neuropathological diagnoses CJD and cerebral vascular disease were not part of this study. *AD* Alzheimer’s disease, *AGD* argyrophilic grain disease, *CBD* corticobasal degeneration, *CJD* Creutzfeldt–Jakob disease, *DLB* Dementia with Lewy bodies, *FTD* frontotemporal dementia, *FTLD* frontotemporal lobar degeneration, *HD* Huntington’s disease, *LBD* Lewy body disease, *MND* motor neuron disease, *MSA* multiple system atrophy, *PD* Parkinson’s disease; *PSP* progressive supranuclear palsy, *VD* vascular dementia

Figure 1 illustrates the relationship between clinical and neuropathological diagnoses.

### Classification metrics of clinical diagnoses

Clinical diagnoses of neurodegenerative diseases exhibited wide spans of sensitivity (0–100%) and positive predictive values (0–96%) depending on the particular disease, while specificity (89.5–100%) and negative predictive values (92–100%) were high for all clinical diagnoses (Table 2).

**Table 2:** Sensitivity, specificity, and predictive values of different clinical diagnoses for the respective correct neuropathological diagnosis in the 455 cases with neurodegenerative diseases in this study

Clinical diagnosis	Sensitivity (95% CI)	Specificity (95% CI)	PPV (95% CI)	NPV (95% CI)
AD	77.3 (69.2–84.1)	89.5 (85.6–92.6)	75.0 (68.3–80.7)	90.6 (87.5–93.0)
AGD	0.0 (0.0–45.9)	100.0 (99.2–100.0)	na	98.7 (98.7–98.7)
CBD	35.0 (15.4–59.2)	97.7 (95.8–98.9)	41.2 (22.9–62.2)	97.0 (96.0–97.8)
CJD	na	98.7 (97.2–99.5)	0.0	100.0
DLB/PD	77.0 (64.5–82.1)	92.6 (89.3–95.1)	74.8 (66.8–81.3)	92.3 (89.7–94.3)
FTD	53.2 (38.1–67.9)	95.3 (92.8–97.2)	56.8 (44.0–68.8)	94.7 (92.9–96.0)
HD	100.0 (84.6–100.0)	99.8 (98.7–100.0)	95.7 (75.6–99.4)	100.0
MND	100.0 (69.2–100.0)	97.8 (95.9–98.9)	50.0 (35.1–64.9)	100.0
MSA	83.8 (68.0–93.8)	98.8 (97.2–99.6)	86.1 (72.0–93.8)	98.6 (97.0–99.3)
PSP	67.5 (55.9–77.8)	97.9 (95.9–99.1)	86.7 (76.3–92.9)	93.67 (91.5–95.3)
VD	na	97.8 (96.0–98.4)	0	100.0

If different clinical diagnoses were made in consecutive work-ups, the accurate diagnosis was taken if made; otherwise the last available diagnosis was used for analysis

*AD* Alzheimer’s disease, *AGD* argyrophilic grain disease, *CBD* corticobasal degeneration, *CI* confidence interval, *CJD* Creutzfeldt–Jakob disease, *DLB* Dementia with Lewy bodies, *FTD* frontotemporal dementia, *HD* Huntington’s disease, *MND* motor neuron disease, *MSA* multiple system atrophy, na not applicable, *NPV* negative predictive value, *PD* Parkinson’s disease, *PPV* positive predictive value, *PSP* progressive supranuclear palsy, *VD* vascular dementia

**Fig. 2:**
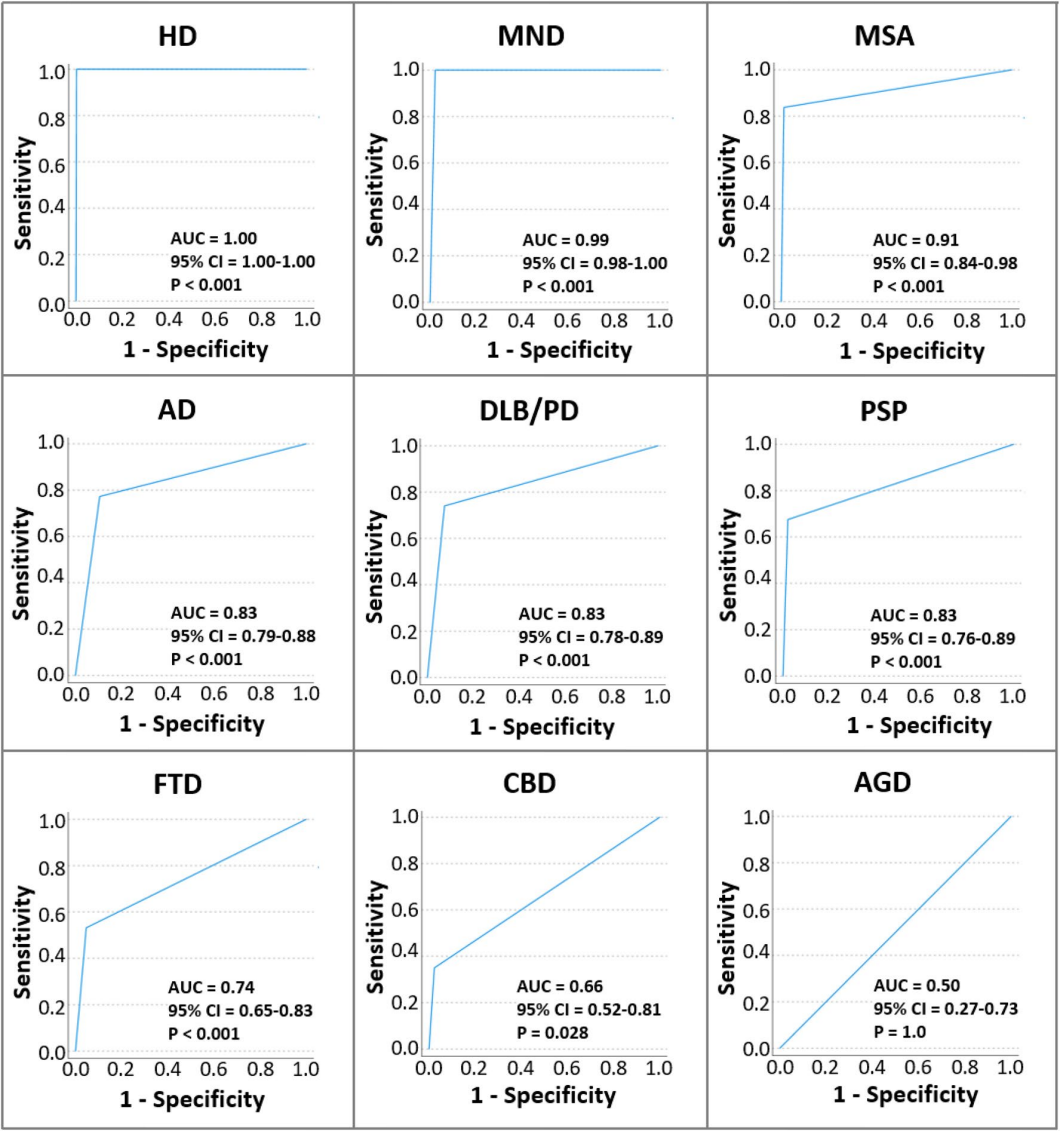
Receiver-operating characteristic (ROC) curves and results from area under the curve (AUC) analysis for different clinical diagnoses including p values for testing the null hypothesis AUC = 0.5. Clinical diagnoses are ordered according to AUC, from best to worst. ROC curves could not be generated for the clinical diagnoses Creutzfeldt–Jakob disease and vascular dementia, since the study population did not include the respective neuropathological diagnoses for reference. *AD* Alzheimer’s disease, *AGD* argyrophilic grain disease, *AUC* area under the curve, *CBD* corticobasal degeneration, *CI* confidence interval, *DLB* Dementia with Lewy bodies, *FTD* frontotemporal dementia, *HD* Huntington’s disease, *MND* motor neuron disease, *MSA* multiple system atrophy, *PD* Parkinson’s disease, *PSP* progressive supranuclear palsy

The overall discriminatory power of clinical diagnoses as determined with AUC analysis was very good (AUC > 0.9) for HD, MND and MSA (*P* < 0.001, respectively), good (AUC = 0.8–0.9) for AD, DLB/PD and PSP (*P* < 0.001, respectively), moderate (AUC = 0.7–0.8) for FTD (*P* < 0.001), limited (AUC = 0.51–0.7) in the *n* = 20 cases with CBD (*P* = 0.03), and no discrimination capacity (AUC = 0.5) in the *n* = 6 cases with AGD (*P* = 1.0) (Fig. 2).

### Time and accuracy of clinical diagnoses

Among all neurodegenerative diseases, the first clinical diagnosis was made on average 3.8 years after onset of the first disease symptom. For the correct clinical diagnosis, it took 4.5 years on average. The fastest correct clinical diagnosis was achieved for MND (after a mean of 1.4 years and at the same time as the first clinical diagnosis), the latest for HD (after a mean of 9.0 years after onset of the first symptom) (Table 1). Taking survival for reference, the on-average fastest correct diagnosis was made for AD (after 41% of survival time) and the latest for CBD (after 82% of survival time).

## Discussion

Clinical diagnoses of neurodegenerative diseases yielded varying diagnostic accuracies when compared to neuropathological diagnosis as the gold standard, reaching from no discriminatory capacity in the *n* = 6 cases with AGD to very good for HD, MND, and MSA. This pattern is broadly consistent with clinical–neuropathological experience, as disorders with relatively stereotyped phenotypes, such as HD and MND, are more readily recognized during life, whereas diagnostic accuracy is lower for entities characterized by marked pathological and clinical heterogeneity, including AGD and CBD. Importantly, however, such experience is typically based on selected cohorts and limited systematic validation; the present study provides a comprehensive, comparative clinicopathological assessment across multiple neurodegenerative disease entities.

In line with the previous studies [2], we observed an overall good discriminatory power for clinical AD diagnoses. A main contributor to the group of false negatives and therefore for lowering sensitivity of clinical diagnosis was a diagnosis of vascular dementia, potentially due to an overestimation of the contribution of vascular lesions to the condition of the patient. The largest portion of patients with a false-positive diagnosis of AD had LBD, probably explained through the in most cases clinical indistinguishable dementia phenotypes of AD and LBD (DLB) and the unavailability of biomarkers with sufficient discriminative power.

Clinical diagnosis of PD or DLB exhibited the same good discriminatory power as a clinical AD diagnosis. A main proportion of false-positive clinical PD diagnosis were found to have an underlying PSP pathology. Another relevant part of clinical PD diagnoses resulted in a neuropathological diagnosis of AD. This may be explained through motor phenotypes in AD [26], or by a predominant AD pathology that may develop in later stages of PD. Most of false-negative cases with a diagnosis of LBD were clinically diagnoses with AD. Clinical diagnoses of PD or DLB were reported to have an 80% accuracy in respective metaanalyses for PD or DLB [3, 4]. However, these metaanalyses are used only in part studies with neuropathological diagnosis as the reference. The clinical diagnosis of the third examined neurodegenerative disease with primary α-synuclein pathology, MSA, showed a very good discriminatory power, in accordance with previous studies [6].

Despite a good discriminatory ability of the clinical diagnosis PSP, the sensitivity of 67.5 was relatively low compared to other diagnoses of neurodegenerative diseases in this study (median 77.0). This may be due to an underdiagnosis of the non-Richardson PSP phenotypes, for which were accounted systematically first in the PSP diagnostic criteria from Höglinger and colleagues in 2017 [17]. An even higher heterogeneity of clinical phenotypes [27] may account for the even lower sensitivity of 35 of the clinical approach to diagnose another neurodegenerative disease with 4R tau pathology, CBD. Not surprisingly, as there are so far no clinical efforts to clinically diagnose the 4R tau disease AGD, sensitivity was 0 and specificity 100, without a discriminatory ability, for the clinical diagnosis of AGD. AGD was reported to present with cognitive and/or behavioral phenotypes [10]. Clinical diagnoses in the six AGD cases of this study were AD (*n* = 2), CBD, FTD, PD, and PSP. These results indicate a necessity for a biomarker to detect AGD.

Regarding the neuropathological diagnosis of FTLD, the clinical diagnosis of FTD showed a moderate discriminatory power with a relatively low sensitivity. A main contributor to this are FTLD cases that are erroneously clinically diagnosed as having AD, maybe because of an underestimation of the frequency of an early memory phenotype, which occurs in approximately 20% of FTLD cases [27].

As expected for genetically based diagnosis, accuracy metrics of the clinical diagnosis HD were excellent throughout. This was also the case for MND, which has no relevant differential diagnosis in the context of neurodegenerative diseases [28].

This study has limitations. In contrast to the standardized neuropathological assessment of this investigation, the included clinical data are subject to the inherent characteristics of real-world data, including a limited standardization of the clinical work-up among different clinical settings. We restricted inclusion to cases autopsied up to 2017 to analyze a closed cohort with complete neuropathological work-up and clinical documentation, and to provide a pre‑molecular‑biomarker baseline before broader clinical uptake of molecular biomarkers and several revised diagnostic criteria implemented from 2017 onward. Therefore, diagnostic accuracy in contemporary biomarker‑enriched practice may be higher. Some neuropathological diagnoses comprised small case numbers (e.g., AGD), and corresponding accuracy estimates are therefore unstable and should be interpreted cautiously. Importantly, mixed neuropathological substrates are common in neurodegeneration and may blur clinico‑pathological correlations. In our cohort, co‑pathologies were recorded but not incorporated into the primary accuracy metrics. Concomitant pathologies (e.g., AD with Lewy body pathology or AD with TDP‑43/LATE‑NC) can shape the clinical phenotype and may contribute both to false-negative and false-positive clinical diagnoses when a single predominant diagnosis is used as the reference. This is particularly relevant for entities such as AGD, which frequently co‑occurs with other age‑associated pathologies. Mixed pathologies have important implications for the development and validation of biomarker-based diagnostic strategies, which may need to detect or account for co-existing pathologies to improve diagnostic accuracy in real-world clinical settings. While vascular lesions can contribute to clinical phenotypes and diagnostic discordance, vascular pathology was not staged systematically [e.g., by vascular cognitive impairment neuropathological guidelines (VCING)] and cases of primary vascular dementia were not a focus of this neurodegenerative proteinopathy‑centered study.

Our findings are highly relevant in the context of an ongoing shift toward molecular biomarker-based diagnostic approaches. In particular, the limited sensitivity observed for several entities underscores the need for biomarkers that directly capture the underlying proteinopathy during life. Blood- and CSF-based biomarkers, molecular PET imaging, and emerging seeding assays hold promise to substantially improve diagnostic accuracy by enabling earlier and more specific identification of the causal pathology. From this perspective, the present results provide an important pre-biomarker benchmark against which future biomarker-enriched diagnostic strategies can be evaluated.

In summary, discriminatory ability of clinical diagnoses of neurodegenerative diseases varied, mostly because of sensitivity. In particular, three clinical diagnosis had relatively low sensitivity. In the clinical diagnoses PSP and CBD, this may be caused by the heterogeneity of clinical phenotypes, and in FTD by underestimation of the relatively frequent early memory phenotype in FTLD. Based on these findings, an increase of sensitivity of clinical diagnoses may be key to further improve correct identification of neurodegenerative diseases for causal treatment. This may gain importance with future advancing therapy development, when sensitivity becomes more relevant for sporadic diseases. Increasing sensitivity of clinical diagnosis may be possible through development and clinical implementation of molecular biomarkers that are able to indicate the causal proteinopathies of neurodegenerative diseases.

## Data Availability

Data are available upon reaonable request.
